# Les hémorragies post coïtales: à propos de 68 cas et revue de literature

**DOI:** 10.11604/pamj.2016.23.131.9073

**Published:** 2016-03-25

**Authors:** Lahssen Boukhanni, Hanane Dhibou, Wafaa Zilfi, Kawtar Iraki Housseini, Yasser Ait Benkeddour, Abderrahim Aboulfalah, Hamid Asmouki, Abderraouf Soummani

**Affiliations:** 1 Service de Gynécologie Obstétrique, Pôle Mère et Enfant, CHU Mohamed VI, Marrakech, Maroc

**Keywords:** Rapport sexuel, traumatisme, hémorragie, Sexual intercourse, trauma, bleeding

## Abstract

L'acte sexuel consenti ou imposé, peut être à l'origine des traumatismes. L'hémorragie post coïtale est un symptôme gynécologique commun. Elle peut révéler de sérieux problèmes. Le but de notre travail est d’étudier le profil épidémiologique, diagnostique et thérapeutique ainsi que les moyens préventifs en cas de déchirure post coïtale. Il s'agit d'une étude prospective, étalée sur deux ans, mené au service de gynécologie obstétrique du CHU Med VI de Marrakech. Nous avons colligé 68 patientes. L’âge moyen est de 27 ans, la majorité des patientes étaient des nullipares soit 89,7% des cas. La moitié des patientes avaient un mariage traditionnel. Le rapport était consentent dans 74% des cas. L'hémorragie génitale a constitue le motif de consultation le plus fréquent soit 98% des cas. Les lésions hyménales ont été retrouvées dans 39% des cas et la lésion siégeait dans le cul de sac postérieur chez 35% des cas. La prise en charge thérapeutique a consisté en une suture chirurgicale chez 97% des cas, associé à une transfusion sanguine chez deux patientes et une abstinence sexuelle pendant minimum deux semaines chez toutes nous patientes. Le contexte social ainsi que le manque d’éducation sexuelle sont souvent incriminé d'où l'intérêt d'une prise en charge psychologique pour prévenir aussi bien le retentissement du traumatisme sur la sexualité que les récidives.

## Abstract (en)

Consensual or imposed sexual act can cause traumas. Post coital bleeding is a common gynecological symptom. It can reveal serious problems. The aim of our study is to analyze the epidemiological, diagnostic and therapeutic profile as well as preventive measures in case of post coital tear. This is a prospective study, over two years, conducted in the Department of Obstetrics and Gynecology at the Center Hospital University Mohammad VI (CHU) in Marrakech. We collected 68 patients. The average age was 27 years. Most of the patients were nulliparous (89.7% of the cases). Half of the patients had traditional wedding. Sexual intercourse was consensual in 74% of the cases. Vaginal bleeding was the most frequent reason for consultation (98% of the cases). Hymenal lesions were found in 39% of the cases and the lesion involved the posterior fornix of the vagina in 35% of the cases. Therapeutic management consisted of a surgical suture in 97% of the cases, associated with blood transfusion in two patients and sexual abstinence of all patients for at least two weeks. The social context as well as the lack of sex education were often highlighted, hence the importance of psychological support to prevent both the impact of traumas on sexuality and the recurrences.

## Introduction

Le saignement post-coïtal est un symptôme gynécologique commun qui pourrait être le premier signe d'une pathologie grave tels que les néoplasies cervicales intra-épithéliales (CIN), le carcinome de col utérin ou une infection à Chlamydia [1]. Mais le plus souvent le saignement post coïtal peut être secondaire à un traumatisme des organes génitaux, et donc il faut penser qu'il peut s'agir d'une agression sexuelle. Les blessures causées par le coït consensuel peuvent être particulièrement difficile de distinguer de ceux trouvés après l'agression sexuelle d'où l'intérêt d'un examen physique complet et d'une enquête étiologique [2]. L'objectif de notre travail est d’étudier le profil épidémiologique, diagnostique et thérapeutique ainsi que les moyens préventifs en cas de déchirure post coïtale.

## Méthodes

Il s'agissait d'une étude transversale prospective de type connaissance et attitude pratique. L’étude s’était déroulée du 1^er^ janvier 2012 au 30 décembre 2013 dans le service de gynécologie et d'obstétrique du CHU de Marrakech. Un questionnaire a été soumis directement aux femmes qui ont été hospitalisées dans nôtre service et qui ont accepté de participer à l’étude après un consentement éclairé. Il a été soumis à un effectif de 68 patientes. Le questionnaire comportait plusieurs items; ils ont concerné l’âge, le statut matrimonial, les conditions des rapports sexuels, le motif de consultation, le bilan lésionnel, le traitement préconisé et l’évolution.

## Résultats

**Figure 1:**
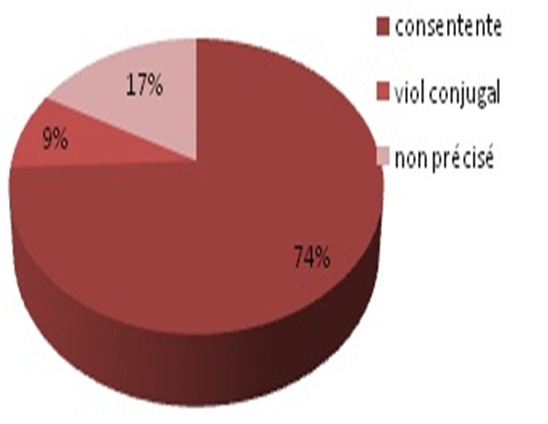
Condition de la relation sexuelle

**Figure 2:**
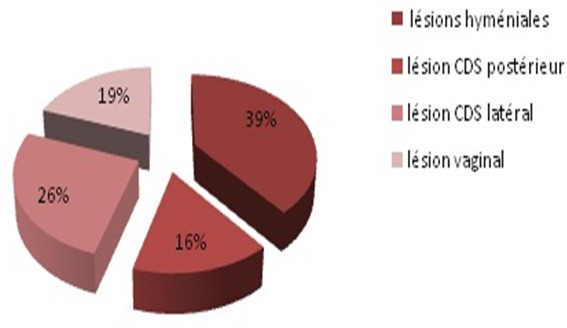
Bilan lésionnel

Nous avons colligé 68 cas sur une période de 24 mois, soit une incidence mensuelle de 2.5. Les patientes étaient âgées de 27 ans en moyenne, paucipares dans plus de 50% des cas, ce traumatisme est survenu plus en période gynécologique (92%) qu'en post partum et notamment dans un contexte de nuptialité (89%). Dans 74% des cas, la relation sexuelle était consentante, alors que dans 9% des cas il s'agissait d'un viol conjugal (Figure 1). Le motif de consultation était l'hémorragie génitale chez 98% des cas. Le délai entre le traumatisme et la consultation variait entre une heure et 3 jours. La lésion vaginale siégeait dans le CDS post (Effet de piston) dans 35% des cas et atteignait au moins 5 cm dans 45% des cas avec un cas flagrant d’éviscération vaginale (Figure 2). Des sutures chirurgicales ont été réalisées dans 97% des cas, associée à une antibiothérapie locale et générale et certes une abstinence sexuelle d'au moins 3 semaines. Le cas d’éviscération a nécessité une laparotomie en extrême urgence.

## Discussion

L'incidence des hémorragies post coïtales varie largement entre les différentes études utilisant une variété de méthodologie. En général, elle est estimée à moins de 1% des urgences gynécologiques [3]. L'utilisation de la colposcopie et des colorations comme le bleu de toluidine a permis le diagnostic des lésions infra cliniques [4]. Dans l’étude de Sommers en 2007, [5] les lésions observées généralement à l'examen direct ont été estimées à 40%, entre 40 et 58% à la coloration par bleu de toluidine, et jusqu’à 87% dans l'examen par colposcopie. Les hémorragies post coïtales pendant les rapports sexuels consensuels sont très rare, ces lésions sont plus fréquentes chez les femmes ménopausées, les adolescentes, les femmes opérées par vois basse ou après une radiothérapie [6]. Une position inhabituelle permettant une pénétration particulièrement profonde de la verge (décubitus dorsal avec hyper flexion des cuisses, position assise), la brutalité ou une hâte excessive sont souvent à l'origine de ces traumatismes [7]. En post partum, les déchirures peuvent être observées lors des accouchements dystociques imposant parfois des manœuvres instrumentales, des déchirures vulvaires accidentelles ou des rétractions cicatricielles des épisiotomies ou des déchirures méconnues ou mal suturées [8]. Dans nôtre études les déchirures post coïtales en post partum ont survenu chez 7% des cas.

Geist note que les hémorragies post coïtales graves sont généralement retrouvées dans les culs de sacs vaginaux, alors que les lésions moins graves sont généralement retrouvées au niveau de la partie inférieure du vagin et de la fourchette vulvaire [9]. Dans nôtre études les lésions des culs de sacs vaginaux (effet de piston) ont été observées dans 42% des cas, et les lésions hyméniales dans 39% des cas avec un cas grave d’éviscération. Lauber et Souma [10] ont également rapporté une différence marquée dans les blessures subies par les rapports sexuels consensuels et non consensuels. Ils ont effectué une comparaison des 22 femmes récemment agressées avec 22 d'autres qui avaient eu des rapports sexuels consentants de pénis-vagin. Neuf (41%) des femmes victimes de violence ont subi des blessures, alors que seulement un (5%) des femmes qui avaient consenti à des rapports sexuels ont subi une blessure. Dans la littérature, de grave cas de déchirures post coïtales ont été rapportés, notamment un cas d’éviscération chez une patiente en péri ménopause pour lequel une cœlioscopie a été réalisée [11]. Egalement, deux cas de déchirures post coïtales graves avec perforation du cul de sac vaginal postérieur et hémopéritoine chez deux jeunes femmes ont été décrits. Un retard diagnostic a été responsable de choc hypovolémique indiquant une laparotomie ainsi qu'une transfusion [3]. Chez les adolescentes, 4 cas ont été décrits avec des lésions vaginales profondes arrivant au cul de sac vaginal postérieur et qui ont nécessité une prise en charge chirurgicale [12].

## Conclusion

L'incidence des hémorragies post coïtales est sous estimée dans nôtre contexte marocain, le contexte social ainsi que le manque d’éducation sexuelle est souvent incriminé d'où l'intérêt d'une prise en charge psychologique pour prévenir aussi bien le retentissement du traumatisme sur la sexualité que les récidives.

### Etat des connaissance sur le sujet

- Le saignement post-coïtal est un symptôme gynécologique commun.
- Il peut être un signe d'une pathologie grave tels que les néoplasies cervicales, mais le plus souvent le saignement post coïtal peut être secondaire à un traumatisme.

### Contribution de notre étude a la connaissance

- Etude du profil épidémiologique, diagnostique et thérapeutique des déchirures post coïtales dans notre contexte
